# Perceptions and Experiences of Developing Prediabetes Awareness and Participating in the National Diabetes Prevention Program Among Hispanic US Adults: Qualitative Study

**DOI:** 10.2196/66964

**Published:** 2025-02-11

**Authors:** Kevin L Joiner, Mackenzie Adams, Edith Beltrán Minehan, Matthew J O'Brien

**Affiliations:** 1 School of Nursing, University of Michigan, 400 N Ingalls St, Ann Arbor, MI, 48109, United States, 1 734-647-0127; 2 School of Public Health, University of Michigan, Ann Arbor, MI, United States; 3 School of Academic Advancement, Madison Area Technical College, Madison, WI, United States; 4 Institute of Public Health and Medicine, Feinberg School of Medicine, Northwestern University, Evanston, IL, United States

**Keywords:** prediabetes, Hispanic, prevention, diabetes, diabetes prevention, awareness, older adult, elder, aging, diabetes prevention program, diabetic, type 2 diabetes, type 1 diabetes, endocrinology, diabetes mellitus, community-based, qualitative study, interview, United States

## Abstract

**Background:**

Prediabetes conveys an increased risk for subsequently developing type 2 diabetes (T2D). The National Diabetes Prevention Program (DPP) is a widely available intensive behavioral intervention that decreases the risk of developing T2D in adults with prediabetes. Data are needed to inform approaches to increase prediabetes awareness and National DPP participation. Few studies have explored perceptions and experiences of prediabetes diagnosis and National DPP participation, and none have focused on Hispanic adults and participation in the National DPP as implemented by a community-based organization.

**Objective:**

This study aims to explore perceptions and experiences of developing prediabetes awareness and participating in the National DPP among Hispanic US adults.

**Methods:**

The sample was recruited from participants in the National DPP as implemented in Spanish by a community-based organization in the upper Midwest. Semistructured interviews were conducted by telephone in April and May 2021. A qualitative descriptive approach was used. Data from the interviews were reviewed, coded, and integrated into themes to reflect the narratives elicited in the interviews.

**Results:**

A total of 16 interviews were conducted. The mean age of the participants was 46 (SD 6, range 34‐55) years. Most (n=15) identified as female. The majority (n=15) reported having been born in Mexico. More than two-thirds (n=11) had a level of educational attainment of high school completion or less. Nearly half (n=7) reported not having health insurance. Qualitative description resulted in the emergence of four main themes: (1) processing the news of having prediabetes, (2) deciding on treatment for T2D primary prevention, (3) valuing language and cultural congruence in the National DPP, and (4) appreciating action-oriented knowledge gained during National DPP participation. Participants described the emotional impact of becoming aware of having prediabetes. National DPP lifestyle coaches’ outreach and recruitment efforts on a local radio program and a Facebook Live (Meta Platforms) broadcast helped raise awareness of prediabetes and influence attitudes toward participation in the National DPP. Values and cultural beliefs appeared to contribute to perceptions and experiences of participating in the National DPP. Participants were inclined to share information about the National DPP with others in their community.

**Conclusions:**

This study presents some of the first evidence exploring perceptions and experiences of developing prediabetes awareness and participating in the National DPP among Hispanic US adults. The findings can inform approaches to increase prediabetes awareness and National DPP participation among Hispanic US adults.

## Introduction

Prediabetes is a state of elevated blood glucose that conveys an increased risk for subsequently developing type 2 diabetes (T2D) [1]. In the United States, approximately 97.6 million adults have prediabetes (38% of the entire population) [2]. Among adults with prediabetes, an estimated 29% will develop T2D within 3 years, and 70% will develop T2D within their lifetime without an effective strategy for primary prevention [3,4]. Lifestyle modification is an effective primary prevention strategy, as demonstrated in a landmark clinical trial of adults with prediabetes and overweight or obesity. Participants randomized to receive the Diabetes Prevention Program (DPP) intensive lifestyle intervention experienced a 58% lower risk of developing T2D over 3 years than the placebo group [5].

Based on this promising evidence, the Centers for Disease Control and Prevention is leading an effort to offer the National DPP, which is derived from the evidence-based intensive lifestyle intervention [6]. The National DPP is implemented in-person and online across the country by community-based organizations, for-profit businesses, health care systems, and insurers [6,7]. The out-of-pocket cost of participating in the National DPP is covered through Medicare, commercial insurance, or employer wellness programs [6]. There are also organizations that enable participation without any out-of-pocket cost through support from philanthropic funds and grants [8].

Despite the high risk that prediabetes portends for subsequent development of T2D and the large-scale effort to provide access to an effective T2D primary prevention strategy, less than 1% of adults with prediabetes have participated in the National DPP [8]. Compared with White non-Hispanic adults with prediabetes, adults with prediabetes of underrepresented racial and ethnic groups are less likely to participate in the National DPP [7,8]. Hispanic adults have the lowest uptake rate into the National DPP among major racial and ethnic groups. Despite making up 15.4% of the US adult population with prediabetes, Hispanic adults only constitute 9% of individuals who have participated in the National DPP [2,8].

Racial and ethnic disparities in National DPP participation are likely multifactorial, stemming from patient-, provider-, and system-level factors. National survey data suggest that only about 20% of adults who meet the glycemic criteria for prediabetes are aware that they have the condition [2]. Even when adults are aware of having prediabetes, evidence suggests that many may not receive counseling from a health care provider about their subsequent risk for developing T2D and the preventive benefits participation in an intensive lifestyle intervention might offer [9]. Factors hindering effective provider-patient discussions about prediabetes and the National DPP include limited educational materials, time constraints during clinic visits, provider perceptions of potential financial constraints to participation, and provider perceptions of patient low self-efficacy for lifestyle change [10-14]. An analysis of 2016 and 2017 National Health Interview Survey data found that less than 5% of adults with diagnosed prediabetes received a referral to the National DPP [15].

Qualitative data from Hispanic adults with prediabetes who have participated in the National DPP are needed to understand their perceptions and experiences about becoming aware of having prediabetes, as well as making an informed choice about initiating and continuing participation in the National DPP. Previous research in the context of T2D prevention in Hispanic adults has explored perceptions regarding the risk of developing diabetes and preferences for prediabetes treatment options [16,17]. In addition, qualitative studies have provided insight into perceptions and experiences of participation in the National DPP as implemented by an integrated health care system among adults with diverse ethnic backgrounds [18,19]. Research in the setting of the National DPP implemented by a community-based organization can augment these earlier qualitative findings.

The objective of this study was to explore perceptions and experiences of prediabetes awareness and National DPP participation among Hispanic adults. The findings from this study will inform future interventions to increase prediabetes awareness and National DPP participation in a high-risk population while helping promote racial and ethnic equity in the prevention of T2D.

## Methods

### Overview

A qualitative descriptive approach was used [20]. This approach is intended to systematically summarize individual participants’ specific and unique perspectives and experiences and report them in detailed descriptions that are closely linked to the words of the participants. Semistructured interviews were used to collect in-depth perspectives and experiences from the participants, allowing their responses to be considered within context [21]. Specifically, follow-up questions were included to elicit more detail about how perspectives and experiences might be influenced by language and cultural factors [22]. The research team was led by the first author, who is a nurse practitioner with a PhD in nursing and formal training and experience in qualitative methods. Additional team members included a research assistant with a PhD in Spanish and a research assistant with a master’s degree in public health, both of whom had experience but no formal training in qualitative methods.

The sample was recruited from among current and completed participants of the National DPP, which was implemented in Spanish by a community-based organization, the National Kidney Foundation of Michigan, in Kent County, an urban county in West Michigan (April and May 2021). According to the US 2020 Census, the population of Kent County, Michigan, was approximately 650,000, with 11.7% of residents identifying as having Hispanic or Latino ethnicity [23].

The eligibility criteria were being 18 years or older, identifying as having Hispanic or Latino ethnicity, having a history of prediabetes, and participating in the National DPP. Participating in the National DPP was defined as attending at least one session. There were no exclusion criteria. One of the lifestyle coaches who facilitated the National DPP in Spanish, distributed study flyers in English and Spanish through email to National DPP participants. Those interested in learning more about the study completed an online screener in which they provided their contact information. Individuals who supplied their contact information in the screener were reached by telephone by the first author, who speaks fluent Spanish and identifies as being non-Hispanic White. The first author was unknown to the participants. Those who agreed to participate were subsequently enrolled in the study.

After obtaining verbal informed consent, the first author offered to conduct the semistructured interview in English or Spanish, depending on the participant’s preference. All of the participants chose to complete their interview in Spanish. Textbox 1 presents the semistructured interview guide in English. Sociodemographic and clinical data collected included age, gender identity, country of birth, English language proficiency, language spoken at home, educational attainment, insurance status, and annual household income.

Textbox 1. **Interview guide.**

Interview questions:

- When did you first learn you had prediabetes?
- Before your diagnosis, what did you know about prediabetes?
- What do you know about the consequences of prediabetes?
- What was your reaction when you were diagnosed?
- Tell me about what it was like when you were diagnosed. How did it make you feel?
- How did you learn about the National Diabetes Prevention Program (DPP)? Did you know someone who had joined?
- Which organization presented the National DPP that you participated in?
- How did your diagnosis influence your decision to participate in the National DPP?
- What made you want to join the National DPP? What gave you motivation?
- If you were to try to convince someone to join the program, what would you tell them?
- Did you encounter obstacles or barriers to participating? Did you have to make any sacrifices to be able to attend? Did you need to find childcare, miss work, or find transportation? How did you overcome these barriers?
- Was there any aspect of the program that made it easier for you to join?
- Which aspects of the National DPP did you like the most or find the most helpful?
- Which aspects of the National DPP did you not like or not find helpful?
- What do you think you learned or gained from the program?

A research assistant, a native Spanish speaker of Mexican heritage who identifies as having Hispanic ethnicity, transcribed the audio recordings of the interviews and translated them from Spanish into English. The research assistant, well-acquainted with the Spanish-language dialects used by the participants, took special care in the translation. The translated transcripts were reviewed by the first author and updated for accuracy when needed. Independently, the research assistant and the other research assistant, who identifies as having non-Hispanic White ethnicity and race, read all of the transcripts and created a list of possible codes with a description for each code. Based on the list of the codes and their descriptions, a codebook was developed by the 2 research assistants and the first author through consensus. The research assistants then independently applied the codes to text segments using ATLAS.ti (Scientific Software Development) computer-aided qualitative data analysis software [24]. The rationale for using computer-aided qualitative data analysis software was to assist in applying the codes and organizing and managing the textual data. As new concepts were identified inductively, additional codes were added to the codebook. After all the transcripts were coded, discrepancies in coding were identified and discussed by the research assistants and the first author. Any differences in coding were adjudicated by the first author and resolved through consensus. Using coding reports, patterns were identified, and a consensus was reached on the themes to be reported. During the analysis, data reduction tables were used to facilitate the iteration of concepts and the development of themes. In addition, audit trails were used to document analytical processes. Thematic saturation was achieved when additional evidence no longer changed the overall description of the themes. Standards for Reporting Qualitative Research endorsed by the EQUATOR (Enhancing the Quality and Transparency of Health Research) Network were followed [25].

### Ethical Considerations

The study was approved by the institutional review Board of the University of Michigan (HUM00192113). Informed verbal consent was obtained from all participants before the interviews were conducted. All transcripts were deidentified during transcription, before analysis. Unique identifiers were used to anonymize quotes from individual participants. Each study participant received a US $20 Amazon gift card as compensation for their time spent participating.

## Results

### Overview

A total of 16 participant interviews were conducted. Table 1 presents the characteristics of the participants. The mean age was 46 (SD 6, range 34‐55). Most (n=15) identified as female. The majority (n=15) reported being born in Mexico. More than two-thirds (n=11) had a level of educational attainment of high school completion or less. Nearly half (n=7) reported not having health insurance.

Qualitative description resulted in the emergence of four main themes: (1) processing the news of having prediabetes, (2) deciding on treatment for T2D primary prevention, (3) valuing language and cultural congruence in the National DPP, and (4) appreciating action-oriented knowledge gained during National DPP participation.

**Table 1.** Participant demographics (N=16).

Characteristic	Values
Age (years), mean (SD; range)	46 (6; 34-55)
**Gender, n**	
Male	1
Female	15
**Country of birth, n**	
Mexico	15
Puerto Rico	1
**English proficiency, n**	
I don’t speak English	3
Not well	11
Well	1
Very well	1
**Language spoken at home, n**	
Spanish	13
Spanish and English	3
**Educational attainment, n**	
Elementary school (grades 1‐8)	2
High school (grades 9‐12)	9
College or graduate school	5
**Insurance status, n**	
Private insurance	3
Public insurance	4
No insurance	7
Annual household income (US $), mean (range)	35,500 (17,000-96,000)

a Not applicable.

### Theme 1: Processing the News of Having Prediabetes

Participants shared a range of unique perceptions and experiences upon becoming aware that they had prediabetes, often through specific events. Commonly, individuals were informed by their health care provider based on the results of a glycated hemoglobin (HbA_1c_) screening test performed during a routine physical exam. Other participants reported developing awareness of having prediabetes based on a point-of-care HbA_1c_ test performed at a health fair. In one instance, a participant discovered her prediabetes following a pregnancy complicated by gestational diabetes:

My prediabetes started… well, 9 years ago. I was pregnant. My son is now 9 years old. Back then, they told me I had gestational diabetes. And after that, I went back to the doctor after giving birth and all of that. Then they told me that I was in the prediabetes [range]. And, I still have prediabetes now.

[Participant 16]

Participants who learned about their prediabetes during a routine physical exam or a visit to a health fair described the emotions they experienced while processing the news of having prediabetes. One woman described how she was caught off guard by the diagnosis of prediabetes during a routine physical examination:

I reacted with, well, surprise and also, a little bit of sadness and anger. Because one thinks they’re okay until they get checked. All the time, every year, I get my physical, and before, it never turned out that way… that I had prediabetes.

[Participant 11]

For some participants, their diagnosis was a chance to begin understanding prediabetes and making lifestyle changes, including attempting to “eat healthier” and “exercise more.” The words of one woman conveyed the sentiment of the participants:

I felt distressed. Sad. But this distress and this sadness, I focused it in a positive direction.

[Participant 6]

Several participants explained that the news of having prediabetes was followed by concern about their future quality of life if they were to develop T2D. Nearly all the participants had at least one close family member with T2D, which informed their views of what it would be like to live with diabetes. For example, one woman explained:

Yes, I knew [about T2D] because my mother-in-law died from it. And a lot of family on my husband’s side [have T2D]. And I knew that they had to inject insulin and check their blood and things like that. I knew a little.

[Participant 6]

In one case, a participant reported that her health care provider outlined a potential future medication scenario for her if she went on to develop T2D:

He told me that I was prediabetic and that if I kept going, the next step would be diabetes, and that I would have to take the medicines that are necessary, and possibly, after that, I would have to take insulin. And that is when I said “No… I don’t want that.” I had to do something with myself so I would not get to the point of having diabetes.

[Participant 2]

### Theme 2: Deciding on Treatment for T2D Primary Prevention

Many participants reported being concerned about the risk of subsequently developing T2D and considering possible paths to T2D primary prevention. The only options presented by the health care provider were typically losing weight through eating healthier meals and being more physically active or taking medication. When asked if the individual who had delivered their prediabetes diagnosis had offered the National DPP or any other support for achieving modest weight loss, a few participants reported that they were referred to a nutritionist. However, most participants reported not being offered any support for healthy lifestyle changes and weight loss. To many, at the time of their prediabetes diagnosis, it seemed that the only option was to try lifestyle changes on their own. One woman described her experience choosing between making lifestyle changes and taking medication:

I first went to my appointment, and they drew blood. Then, later, I came back for the results. It was then that the doctor told me what was going on and that I should try to get better, lose weight, and eat healthier. Because the doctor knows I am very reluctant to take medicines. I said: “No, I will do it on my own, lose weight, and do it.

[Participant 2]

One woman explained:

It was not my doctor [from who I heard about the National DPP]. My doctor would only say: “You need this [to eat healthier and lose weight]. You need that [to take a medication].” She never told me that there is a diabetes prevention program.

[Participant 1]

Participants expressed that the prospect of having to eat healthier and lose weight on their own, without support, seemed overwhelming. One woman shared her frustration:

I want to lose weight, but I cannot. Meanwhile, for example, during Holy Week, my clothes did not fit me, and I thought: What can I do? Sometimes, I do not have money to buy new clothes, and it’s very unflattering.

[Participant 7]

Another woman stated:

You see that we want to lose weight, and we try to diet. But that only lasts, what, 15 days to a month.

[Participant 8]

The majority of participants learned about the National DPP through public health campaigns after being diagnosed with prediabetes. For example, most participants reported finding out about the National DPP through a local Spanish-language program on the radio or a local broadcast on Facebook Live in which a media personality spoke with a local lifestyle coach. What was immediately appealing to many study participants was how the lifestyle coach prepared them for initiating and participating in the National DPP. The lifestyle coach discussed information about the National DPP that was essential so that they could make an informed choice about participation, including that it would teach them about topics such as “how to eat healthy food” and “how to count calories.” The lifestyle coach also discussed how the National DPP was a free, group-based program. One woman recalled her experience finding out about the National DPP:

I heard about it [the National DPP] on the radio. It was on the program La Poderosa. She [the local lifestyle coach] was saying that peoples’ bodies are becoming larger because we have bad habits. And … what caught my attention was that it [T2D] is caused by the decisions that people make … and that people need counseling, some motivation. And I said: “Oh, that sounds interesting!” … I wrote down the number, called, and registered myself.

[Participant 6]

Another woman described her experience finding out about the program:

I responded to the video that she [the lifestyle coach] made on El Informador [Facebook Live program]. She said that she was going to teach us how to take care of ourselves better so that we wouldn’t develop it [T2D]. And we would be changing our lifestyle and eating healthier. And that she would support us.

[Participant 8]

In addition, a couple of participants who became aware that they had prediabetes at a health fair reported that the National DPP had been offered to them as an opportunity for T2D primary prevention.

### Theme 3: Valuing Language and Cultural Congruence in the National DPP

Participants reported that an important factor in their engagement with the National DPP was the linguistic and cultural tailoring for Hispanic adults. The fact that the program was in Spanish was described as essential. In addition to the language, participants expressed their appreciation that the program was culturally salient in other ways. One participant shared that the National DPP offered her access to information that she felt that she was missing from visits with her health care provider. She expressed that this was especially important since many Hispanic women like herself may not visit a health care provider to manage their health. She explained:

To tell you the truth, as a Hispanic, you take things differently. One does not go to the doctor just because. You go to the doctor when you are sick and when you feel bad. You do not go to get a physical or anything. So, I learned a lot in it [the National DPP] about what you should look for in the labels. What you must do and all that, I learned it there.

[Participant 11]

When considering how being Hispanic might present unique challenges or opportunities to engage with the National DPP, one woman identified her country of origin as impacting her relationship with food choices. She explained:

I’m Mexican, and we are used to eating… no not eating, ingesting, too much. We’re accustomed to beans, rice, meat… steak, chicken, hamburgers, or whatever it may be, …. and always tortillas. We ingest a lot of carbohydrates. … And one eats and eats, and one doesn’t pay attention to how much one eats. What I learned from the women [the lifestyle coaches] has been of great importance. I’ve learned a lot. Thank God.

[Participant 12]

Some participants perceived that being an immigrant created challenges related to gaining weight and eating behaviors. One woman explained:

I should not gain any more weight because it is very easy here [in the US] to gain weight. That was the only thing I thought. That I won’t gain weight.

[Participant 14]

Another woman explained:

Because the problems of Hispanic people are different from the problems of people who were born here. Having left your country, the pain of leaving your parents, your culture, all of that, is painful, and that also causes depression and not eating appropriately.

[Participant 6]

### Theme 4: Appreciating Action-Oriented Knowledge Gained During National DPP Participation

Many participants appreciated the action-oriented knowledge they gained during National DPP participation, which promoted their attempts at healthy lifestyle change. One woman explained:

From the beginning, when we started with the teacher, she taught us that we need to be detectives of nutrients and food labels. I had never paid attention to that before. I had tried to look for things with less sugar and stuff, but never stopped to look at ingredient by ingredient and the amount of fat. This is what we also studied in class. I tell you, for me, that was something new… She also taught us about exercise, and she showed us what we could do at home.

[Participant 7]

Another woman stated:

I’ve seen nutritionists before, and I was the type of person who wanted to be told, ‘Ok, in the morning, you will eat this; at noon, you will eat that; and at night, you will eat this.’ But no one tells you that … She [the lifestyle coach] is teaching us how to read food labels to find out about calories, sugars, and fats.

[Participant 8]

A participant recalled the early challenges of learning about prediabetes and reported that understanding biological mechanisms offered in the National DPP enhanced his motivation to make healthy lifestyle changes. He noted:

For me, at first, it was very complicated. But then after that, when relating how one eats to how sugars rise, and knowing that the pancreas regulates sugars, it’s then when one starts to understand why, before eating and after eating, one’s sugar levels are different.

[Participant 11]

Participants discussed how they perceived that they were able to carry over the focus on the empowerment of the National DPP to other family members.

To tell you the truth, it [the National DPP] did help me because I have … small children. … I do not want what happened to me to happen to them. I mean, it [the National DPP] helps you. It helps you. It helps make your family better. They [the lifestyle coaches] help you to take care of your family.

[Participant 3]

Some of the participants seemed to feel confident sharing the knowledge that they gained from the National DPP with others in the community. One woman recounted:

In my experience, I have gotten to know more people and told them, when they say “prediabetes,” you have to take it seriously…and try to change your way of living and lifestyle.

[Participant 3]

Another woman explained:

There should be more, like, more programs. Like more publicity. Give us more information.… They should give programs like this [the National DPP] more promotion. Also, tell people more so they know about it. Because the truth is, one doesn’t know about them. Look after this, the truth is that I sent a lot of people there. I told them: “Go. Go. It’s very important.”

[Participant 1]

## Discussion

### Principal Findings

This qualitative study presents some of the first evidence exploring perceptions and experiences of developing prediabetes awareness and participating in the National DPP among Hispanic US adults. The findings emphasize the emotional impact of becoming aware of having prediabetes and highlight the significance of dynamic outreach and recruitment by National DPP lifestyle coaches. Outreach by National DPP lifestyle coaches, who leveraged local community forums, including a local radio program and a Facebook Live broadcast, was pivotal in raising awareness of prediabetes and influencing attitudes toward participation in the National DPP. Values and cultural beliefs among US Hispanic communities appeared to contribute to perceptions and experiences of participating in the National DPP. Some participants expressed a strong inclination to share information about the National DPP with others in their community.

Participants vividly remembered the moment they found out that they had prediabetes and the emotions evoked by the diagnosis. Emotions such as surprise, distress, and concern about their future quality of life were commonly reported. This resonates with findings in other communities where similar emotional reactions to developing prediabetes awareness have been documented [26]. The familiarity with T2D and the way T2D had disrupted the lives of close family members and friends was also consistently and clearly communicated, which complements previous research on culturally relevant issues among Hispanic adults in the United States [27]. When reflecting on their views before joining the National DPP and how participating had affected them, the participants highlighted a previous lack of knowledge regarding prediabetes and a previous feeling of lack of control over their trajectory toward developing T2D. For many, the diagnosis of prediabetes served as a catalyst for participating in the National DPP and taking proactive measures such as healthier eating and increased physical activity. These findings highlight the importance of providing clear, easily understood, complete information to enhance individuals’ optimism regarding the risk that prediabetes portends for developing T2D and the effectiveness of the National DPP in mitigating this risk.

Guidelines recommend primary care providers play an essential role in T2D primary prevention through prediabetes screening, counseling, and support for initiating National DPP participation [28,29]. However, the findings suggest that primary care providers often fail to effectively counsel Hispanic adults with prediabetes about their risk for developing T2D and the benefits of the National DPP [9,15]. This gap could be attributed to limited awareness among primary care providers about the National DPP and the community-based organizations implementing it [13,14]. Participants echoed this concern, indicating that many did not learn about the National DPP from their health care providers but through other channels.

The role of trusted community figures and effective communication through local media emerged as a key factor in participants’ decisions to join the National DPP. Information disseminated through appearances by lifestyle coaches on a local Spanish-language radio program, and a Facebook Live broadcast provided essential insights into the National DPP, making it accessible and appealing. This aligns with previous research that highlights the positive impact of social media on health promotion and National DPP recruitment, particularly within Hispanic communities where the use of social media is prevalent [30,31].

Participants valued the National DPP implemented by the community-based organization for its linguistic and cultural congruence, noting that it resonated with their Hispanic heritage. Cultural factors like traditional diets and the challenges faced by immigrants in adopting healthier lifestyles were common themes. The National DPP’s emphasis on action-oriented knowledge, teaching participants about reading food labels, and understanding the biological mechanisms of prediabetes, empowered them to make informed health choices. This sense of control that they gained extended beyond their own personal health, influencing family members and potentially enhancing health consciousness in the community.

Participants’ appreciation of the National DPP extended to its broader impacts on family and community. Promotion through family and friends was also highlighted as a valuable channel for reaching more people, including men who might be hesitant about participating or facilitating their spouse’s participation. Participants suggested that more supportive efforts and more comprehensive promotion could help inform others in their community about prediabetes and combat fatalism, which was viewed as a cultural belief that might have an impact on how treatments such as the National DPP for primary prevention of T2D are viewed [32].

Offering the National DPP at no out-of-pocket cost helped to alleviate socioeconomic barriers, making the program accessible to those with fewer resources [33]. Lifestyle coaches marketing directly to adults with prediabetes could help overcome potential financial concerns that health care providers may have regarding patient National DPP participation [34]. This approach holds promise for reaching underserved communities and warrants further exploration of coordinated interventions between health care systems and community outreach.

### Limitations

This study has several limitations. The Hispanic adults with prediabetes who took part in the study had participated in the National DPP as implemented by a community-based organization, which was in Spanish and culturally tailored for the Hispanic community. Therefore, this study does not explore perceptions and experiences of Hispanic adults with prediabetes when the National DPP is not accessible in the community in a linguistically and culturally tailored manner. The community-based organization was supported by philanthropic and grant funding, which allowed Hispanic adults with prediabetes to participate in the National DPP without any out-of-pocket cost. This study included an overrepresentation of women and individuals who identify as Mexican American compared with the overall Hispanic adult population in the United States [35]. The overwhelming majority of all National DPP participants (75%) nationwide are women [8]. Notwithstanding these limitations, the qualitative description offers clinicians, researchers, and policymakers, insight into the perceptions and experiences of an at-risk population currently underserved by the National DPP, upon which future studies may expand. Further research in this area should consider sampling from a larger number of National DPP sites in geographically dispersed areas. Expanded representation of adults of diverse racial, ethnic, and cultural backgrounds, as well as male adults, who likely have unique needs and challenges related to being reached and enrolled in the National DPP, are also opportunities for future research.

### Future Directions

A community-based intervention for Hispanic adults that strengthens prediabetes awareness and provides counseling about participating in the National DPP may increase uptake into the National DPP. The American Diabetes Association and the Centers for Disease Control and Prevention advocate for patient-centered approaches in primary care to strengthen awareness of prediabetes and support decisions about National DPP participation [6,29]. The potential for local communication channels, including radio programs and social media broadcasts, to amplify health care services’ National DPP recruitment and enrollment efforts at scale is also important [36]. In total, 66% of Hispanic adults use Facebook [31,37]. Having an interview with a National DPP lifestyle coach on a radio program or a broadcast on Facebook Live leverages community forums and requires much less investment of resources than health care services calling many people to ask them to join the National DPP [38]. Interventions that foster joint efforts through multiple communication channels have the potential to make the path from prediabetes diagnosis to National DPP participation smoother and more effortless and reach underrepresented racial and ethnic groups [30].

### Conclusions

Efforts to increase National DPP participation among Hispanic adults with prediabetes should be tailored to these populations’ unique social, cultural, and linguistic needs. This study is helpful for primary care providers, community organizations, and researchers aiming to boost prediabetes awareness and National DPP participation. Participants in this study described their perceptions and experiences of being diagnosed with prediabetes and what they gained from participating in the National DPP, which provides insights for future efforts to advance T2D primary prevention. Overall, participants reported finding the National DPP valuable and well-suited to their needs. First, the enrollment information should be a multipronged effort, including coming through respected, vibrant communication forums that serve the needs of the community. Second, a National DPP lifestyle coach who is a community member would be trusted easily; however, if a lifestyle coach would like to be helpful to a Hispanic community, there must be an awareness of the cultural, social, and linguistic issues pertaining to this community. Finally, it is important to note that the empowerment that the National DPP brings to individuals and families has its core in the community. Empowerment comes from managing the diagnosis of prediabetes with lifestyle changes that affect and impact family and loved ones. Hispanic adults with prediabetes who participate in the National DPP don’t just decrease their own risk for developing T2D; they also become passionate and engaged leaders who have the potential to improve others’ lives in the community.

## Supplementary material

10.2196/66964 Checklist 1 Standards for Reporting Qualitative Research (SRQR) checklist.

## Abbreviations

DPP: Diabetes Prevention Program
EQUATOR: Enhancing the Quality and Transparency of Health Research
HbA_1c_: glycated hemoglobin
T2D: type 2 diabetes

## References

[1] Tabák AG, Herder C, Rathmann W, Brunner EJ, Kivimäki M (2012). Prediabetes: A high-risk state for diabetes development. Lancet.

[2] (2024). National Diabetes Statistics Report 2024. Centers for Disease Control and Prevention.

[3] Nathan DM, Davidson MB, DeFronzo RA (2007). Impaired fasting glucose and impaired glucose tolerance: implications for care. Diabetes Care.

[4] Gerstein HC, Santaguida P, Raina P (2007). Annual incidence and relative risk of diabetes in people with various categories of dysglycemia: A systematic overview and meta-analysis of prospective studies. Diabetes Res Clin Pract.

[5] Knowler WC, Barrett-Connor E, Fowler SE (2002). Reduction in the incidence of type 2 diabetes with lifestyle intervention or metformin. N Engl J Med.

[6] (2024). National Diabetes Prevention Program. Centers for Disease Control and Prevention.

[7] Cannon MJ, Ng BP, Lloyd K, Reynolds J, Ely EK (2022). Delivering the National Diabetes Prevention Program: Assessment of enrollment in in-person and virtual organizations. J Diabetes Res.

[8] Gruss SM, Nhim K, Gregg E, Bell M, Luman E, Albright A (2019). Public health approaches to Type 2 diabetes prevention: the US National Diabetes Prevention Program and beyond. Curr Diab Rep.

[9] Cunningham A, Li E, Silverio A, Mills G, Han J, Waters A (2022). Patient and provider prediabetes knowledge, attitudes, and behavior in a large urban family medicine practice. Ann Fam Med.

[10] Madrigal L, Haardörfer R, Kegler MC (2023). A structural equation model of CFIR inner and outer setting constructs, organization characteristics, and national DPP enrollment. Implement Sci Commun.

[11] Papalii M, Ely E (2023). 333-OR: What motivates people to enroll in the National Diabetes Prevention Program Lifestyle Change Program. Diabetes.

[12] AuYoung M, Moin T, Richardson CR, Damschroder LJ (2019). The Diabetes Prevention Program for Underserved Populations: A brief review of strategies in the real world. Diabetes Spectr.

[13] Tseng E, Greer RC, O’Rourke P (2019). National Survey of Primary Care Physicians’ Knowledge, Practices, and Perceptions of Prediabetes. J Gen Intern Med.

[14] Tseng E, Greer RC, O’Rourke P (2017). Survey of primary care providers’ knowledge of screening for, diagnosing and managing prediabetes. J Gen Intern Med.

[15] Ali MK, McKeever Bullard K, Imperatore G (2019). Reach and use of Diabetes Prevention Services in the United States, 2016-2017. JAMA Netw Open.

[16] Joiner KL, Sternberg RM, Kennedy CM, Fukuoka Y, Chen JL, Janson SL (2016). Perception of risk for developing diabetes among foreign-born Spanish-speaking US Latinos. Diabetes Educ.

[17] O’Brien MJ, Moran MR, Tang JW (2016). Patient perceptions about prediabetes and preferences for diabetes prevention. Diabetes Educ.

[18] Ritchie ND, Phimphasone-Brady P, Sauder KA, Amura CR (2021). Perceived barriers and potential solutions to engagement in the National Diabetes Prevention Program. ADCES in Practice.

[19] Harrison CR, Phimphasone-Brady P, DiOrio B (2020). Barriers and facilitators of National Diabetes Prevention Program engagement among women of childbearing age: a qualitative study. Diabetes Educ.

[20] Sandelowski M (2000). Whatever happened to qualitative description?. Res Nurs Health.

[21] DeJonckheere M, Vaughn LM (2019). Semistructured interviewing in primary care research: A balance of relationship and rigour. Fam Med Com Health.

[22] (2022). Hispanic Americans’ trust in and engagement with science. Pew Research Center.

[23] U.S. Census Bureau (2024). Quick Facts: Kent County, Michigan.

[24] Friese S (2019). Qualitative Data Analysis with ATLAS.ti.

[25] O’Brien BC, Harris IB, Beckman TJ, Reed DA, Cook DA (2014). Standards for reporting qualitative research: a synthesis of recommendations. Acad Med.

[26] Skoglund G, Nilsson BB, Olsen CF, Bergland A, Hilde G (2022). Facilitators and barriers for lifestyle change in people with prediabetes: A meta-synthesis of qualitative studies. BMC Public Health.

[27] Caban A, Walker EA (2006). A systematic review of research on culturally relevant issues for Hispanics with diabetes. Diabetes Educ.

[28] Pronk NP, Remington PL, Community Preventive Services Task Force (2015). Combined diet and physical activity promotion programs for prevention of diabetes: Community preventive services task force recommendation statement. Ann Intern Med.

[29] ElSayed NA, Aleppo G, Bannuru RR (2024). 3. Prevention or Delay of Diabetes and Associated Comorbidities: Standards of Care in Diabetes—2024. Diabetes Care.

[30] Jalkanen K, Järvenpää R, Tilles-Tirkkonen T (2021). Comparison of communication channels for large-scale type 2 diabetes risk screening and intervention recruitment: empirical study. JMIR Diabetes.

[31] (2024). Americans’ social media use. Pew Research Center.

[32] Moreira T, Hernandez DC, Scott CW, Murillo R, Vaughan EM, Johnston CA (2018). *Susto, Coraje, y Fatalismo*: Cultural-bound beliefs and the treatment of diabetes among socioeconomically disadvantaged Hispanics. Am J Lifestyle Med.

[33] (2024). Improving access to diabetes prevention. Center for Disease Control and Prevention.

[34] Thomas TW, Golin CE, Kinlaw AC (2022). Did the 2015 USPSTF abnormal blood glucose recommendations change clinician attitudes or behaviors? A mixed-method assessment. J Gen Intern Med.

[35] (2024). Hispanic/Latino health. U.S. Department of Health and Human Services Office of Minority Health.

[36] Mora J, Romo R, Dempsey S (2023). Engaging the community served: A U.S. Cancer Center’S Facebook live cancer awareness campaign for Spanish-speaking Latinos during COVID-19. Cancer Causes Control.

[37] (2021). Social media use in 2021. Pew Research Center.

[38] Philis-Tsimikas A, Fortmann A, Lleva-Ocana L, Walker C, Gallo LC (2011). Peer-led diabetes education programs in high-risk Mexican Americans improve glycemic control compared with standard approaches: A Project Dulce promotora randomized trial. Diabetes Care.

